# Nipple-coring and purse-string suture technique – A modification of the nipple-sparing risk-reducing mastectomy

**DOI:** 10.1016/j.jpra.2020.06.005

**Published:** 2020-07-09

**Authors:** L. Kearney, E.C. Francis, N. Relihan, E. O'Broin

**Affiliations:** a Department of Plastic and Reconstructive Surgery, Cork University Hospital, Cork, Ireland; b Department of Breast Surgery, Cork University Hospital, Cork, Ireland

**Keywords:** Breast reconstruction, Nipple-sparing mastectomy, Surgical technique

## Introduction

The availability of genetic testing for BRCA-1 and BRCA-2 has revealed many women who are at significantly increased risk of developing breast cancer. This has prompted increased numbers of prophylactic mastectomies, considered to offer patients a risk-reduction of breast cancer as high as 90%.^1^ Over the last 20 years risk –reducing mastectomies with preservation of the nipple-areolar complex are in increasing demand within this patient group. Risk-reducing mastectomy techniques have proven not to cause a compromise in oncological safety,^2^ have resulted in improved cosmesis and also have a suggested reduced overall psychological impact on patients,^3^

Despite this evidence, there is still a level of concern regarding complete oncological safety of preserving the entire nipple-areolar complex.^4^ In addition NAC necrosis is a well-described complication in **nipple-sparing** mastectomies. In an attempt to address these issues two-stage surgical approaches are gaining favour, where the nipple and sub-areolar are undermined to improve peripheral blood supply. The mastectomy is performed as a delayed procedure typically three weeks later. This technique is suggested to be superior in terms of maintaining NAC viability but also allows a biopsy of retroareolar tissue to be sent for histology before proceeding with **the nipple-sparing** mastectomy.^5^ However the additional costs, time and use of resources can make it less desirable for both surgeons and patients than a one-stage procedure.

In order to address these issues, our unit offers a one-stage subcutaneous mastectomy via an inframammary (IMF) incision but cores the central ductal tissue of the nipple. **This results in a modified areolar sparing mastectomy with preservation of an additional cuff of dermis from the nipple.** The nipple defect is then reconstructed with internal purse string sutures. The removal of ductal tissue may potentially confer additional oncological safety but our primary reason for this technique is to provide a satisfactory aesthetic result and eliminates the need for the patient to undergo a secondary procedure. In addition there may be a theoretical reduced risk of implant infection with this technique. The ductal tissue of the nipple has the highest concentration of endogenous breast bacteria, known to be associated with implant contamination and subsequent infection.^6^

## Technique

**Figure 1:**
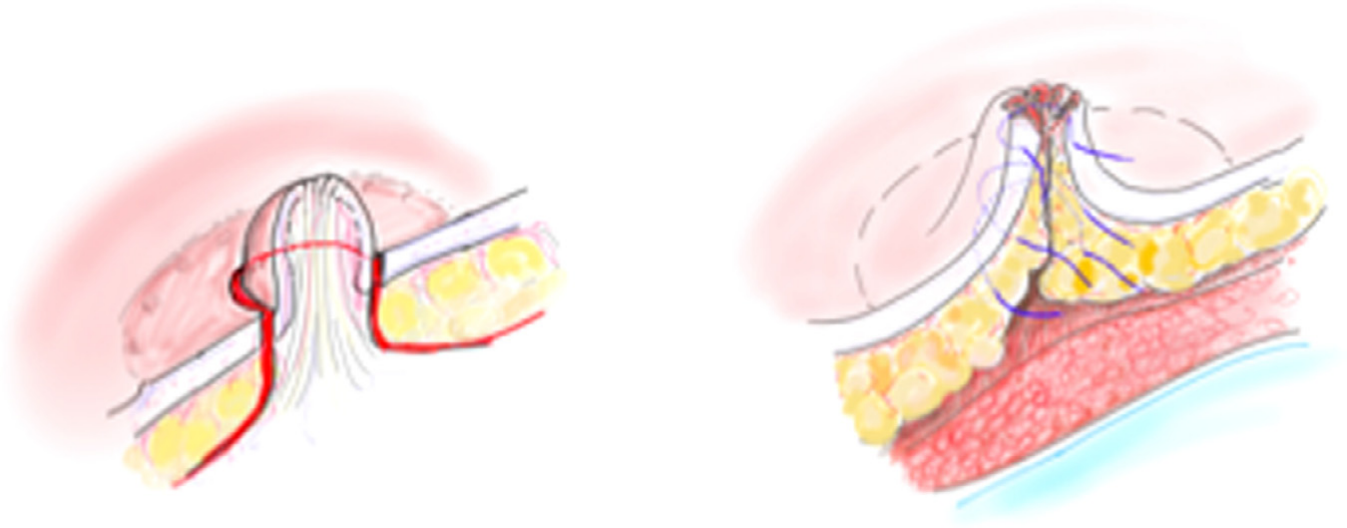
Illustrations of a modified areolar sparing mastectomy with central core of nipple excised and subsequent multi-level internal purse-string sutures.

**Figure 2:**
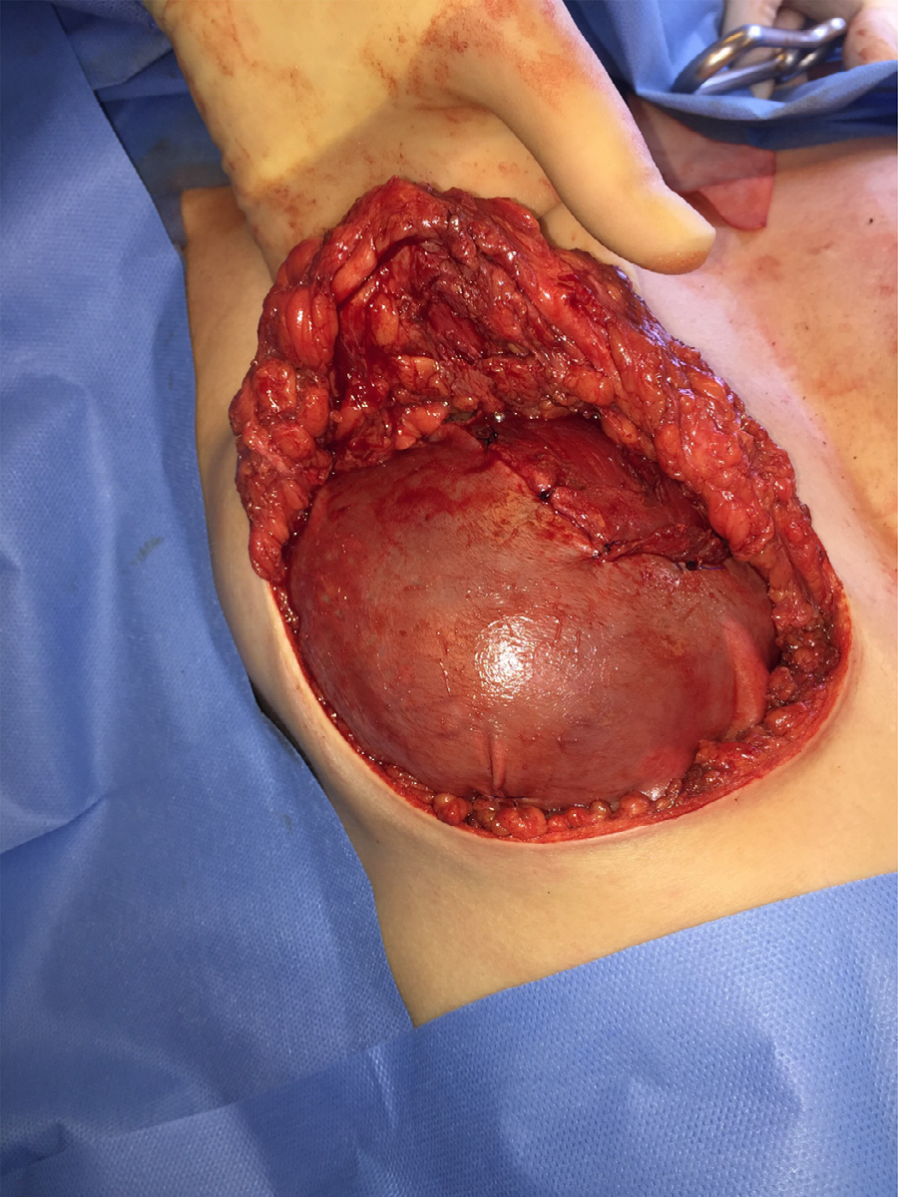
Right subcutaneous mastectomy via IMF incision with central core of nipple excised, submuscular implant placement with Veritas^Ⓡ^) covering lower pole.

**Figure 3:**
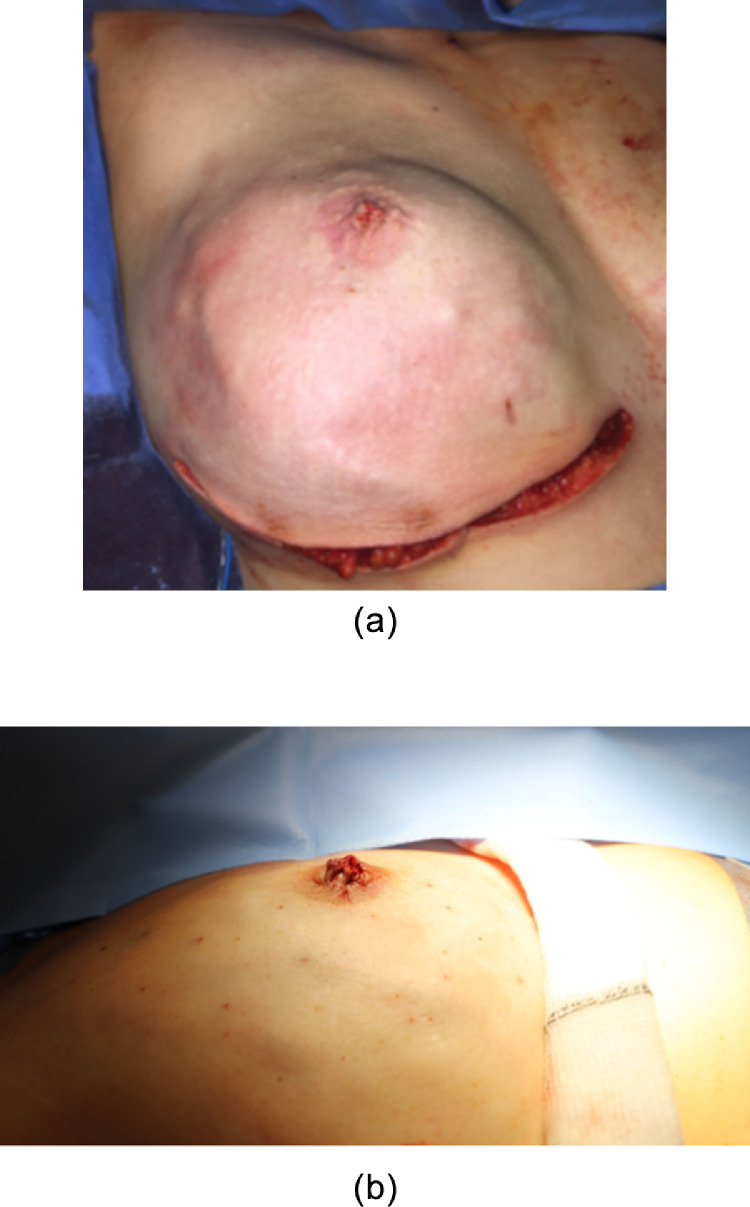
(a): Right subcutaneous mastectomy and resulting neonipple from coring and purse string suture technique (b): Lateral view of nipple reconstruction.

Pre-operatively the inframammary folds are marked in an upright position. Symmetrical incisions along the IMF are made ranging from 8 to 10 cm. A standard subcutaneous mastectomy is performed, leaving adequate skin flap thickness. A ‘coring’ incision is made circumferential around the mid-portion of the nipple. **No ductal tissue is left behind however a cuff of dermis from the base of the nipple is preserved with the areola**. The top of the nipple and its ducts fall back with the breast specimen as the skin flap is lifted off the breast. The resulting circular defect is closed with three 4.0 monocryl purse string sutures placed, in a multilevel fashion in the subareolar skin flap [Figure 1]. The silicone implant is inserted in the subpectoral pocket and a 6 cm × 16 cm piece of bovine pericardium (Veritas**^Ⓡ^)** is sutured to the released inferior pectoralis muscle, to cover the lower pole. The free edge of the collagen matrix is secured laterally and inferiorly to recreate the IMF [Figure 2]. The subcutaneous tissues are closed in two layers with interrupted 3.0 monocryl sutures and a final subcuticular 4.0 monocryl layer is used to close the skin. Percutaneous 5.0 vicryl rapide sutures are used to complete the nipple reconstruction [Figure 3(a) and (b)].

To date the senior authors have performed 8 cases, 4 bilateral with no skin or areolar necrosis. Projection was poor in the first few cases. However, there was an illusion of a nipple, which was very satisfactory to the patients. Increasing projection at a later date using conservative nipple reconstructive techniques, (mini-C-V flaps) is very simple to do. In the last 3 cases more careful coring technique and additional cerclage/purse string sutures have resulted in improved projection at 6 months.

In addition to direct-to-implant procedures this technique could similarly be considered in the setting of tissue expansion placement. The IMF incision is the favoured option in our unit for these reconstructions for reasons previously described, patient and surgeon satisfaction^7^ and low rates of nipple necrosis.^8^ We do not have experience using the nipple-coring and purse-string suture technique with other incisions i.e. radial or periareolar/cirucmareolar approaches. The latter would be expected to have a potential affect on perfusion of residual nipple.

As the demand for risk-reducing mastectomies is expected to continue, the authors feel this a useful technique that can be considered to provide a satisfactory aesthetic result and reduce the need for secondary procedures.
